# Dissecting the pathobiology of altered MRI signal in amyotrophic lateral sclerosis: A *post mortem* whole brain sampling strategy for the integration of ultra-high-field MRI and quantitative neuropathology

**DOI:** 10.1186/s12868-018-0416-1

**Published:** 2018-03-13

**Authors:** Menuka Pallebage-Gamarallage, Sean Foxley, Ricarda A. L. Menke, Istvan N. Huszar, Mark Jenkinson, Benjamin C. Tendler, Chaoyue Wang, Saad Jbabdi, Martin R. Turner, Karla L. Miller, Olaf Ansorge

**Affiliations:** 1 Nuffield Department of Clinical Neurosciences, University of Oxford, Oxford, UK; 2 Wellcome Centre for Integrative Neuroimaging, FMRIB, Nuffield Department of Clinical Neurosciences, University of Oxford, Oxford, UK; 3 Department of Radiology, University of Chicago, Chicago, IL USA

**Keywords:** Amyotrophic lateral sclerosis, Magnetic resonance imaging, *Post mortem* brain, Systematic sampling, Histology, MRI-histology correlation

## Abstract

**Background:**

Amyotrophic lateral sclerosis (ALS) is a clinically and histopathologically heterogeneous neurodegenerative disorder, in which therapy is hindered by the rapid progression of disease and lack of biomarkers. Magnetic resonance imaging (MRI) has demonstrated its potential for detecting the pathological signature and tracking disease progression in ALS. However, the microstructural and molecular pathological substrate is poorly understood and generally defined histologically. One route to understanding and validating the pathophysiological correlates of MRI signal changes in ALS is to directly compare MRI to histology in *post mortem* human brains.

**Results:**

The article delineates a universal whole brain sampling strategy of pathologically relevant grey matter (cortical and subcortical) and white matter tracts of interest suitable for histological evaluation and direct correlation with MRI. A standardised systematic sampling strategy that was compatible with co-registration of images across modalities was established for regions representing phosphorylated 43-kDa TAR DNA-binding protein (pTDP-43) patterns that were topographically recognisable with defined neuroanatomical landmarks. Moreover, tractography-guided sampling facilitated accurate delineation of white matter tracts of interest. A digital photography pipeline at various stages of sampling and histological processing was established to account for structural deformations that might impact alignment and registration of histological images to MRI volumes. Combined with quantitative digital histology image analysis, the proposed sampling strategy is suitable for routine implementation in a high-throughput manner for acquisition of large-scale histology datasets. Proof of concept was determined in the spinal cord of an ALS patient where multiple MRI modalities (T1, T2, FA and MD) demonstrated sensitivity to axonal degeneration and associated heightened inflammatory changes in the lateral corticospinal tract. Furthermore, qualitative comparison of R2* and susceptibility maps in the motor cortex of 2 ALS patients demonstrated varying degrees of hyperintense signal changes compared to a control. Upon histological evaluation of the same region, intensity of signal changes in both modalities appeared to correspond primarily to the degree of microglial activation.

**Conclusion:**

The proposed *post mortem* whole brain sampling methodology enables the accurate intraindividual study of pathological propagation and comparison with quantitative MRI data, to more fully understand the relationship of imaging signal changes with underlying pathophysiology in ALS.

**Electronic supplementary material:**

The online version of this article (10.1186/s12868-018-0416-1) contains supplementary material, which is available to authorized users.

## Background

Amyotrophic lateral sclerosis (ALS) is a typically rapidly progressive, fatal neurodegenerative disorder that is genetically and phenotypically heterogeneous. It is primarily characterised by selective degeneration of upper and lower motor neurons [1]. A significant proportion of ALS patients develop cognitive impairment within the spectrum of frontotemporal dementia (FTD) [2]. The diagnosis is essentially clinical [3]. Conventional magnetic resonance imaging (MRI) is generally used during diagnosis as a tool to exclude ALS mimics, but advanced techniques such as functional MRI and diffusion tensor imaging (DTI) have enabled investigation of both structural and functional connectivity in vivo [4]. Diffusion tensor imaging in particular has highlighted the widespread cerebral pathology associated with ALS [5]. Furthermore, quantitative susceptibility mapping has demonstrated potential as a biomarker of upper motor neuron dysfunction in ALS [6–8]. However, these MRI markers are non-specific and generally known to be influenced by several aspects of tissue related to neuropathology. It is therefore crucial to define the microstructural and molecular pathologic correlates of these MRI measures in ALS. Therefore, MRI-histology correlation analysis in *post mortem* tissue offers a platform to discover the histological underpinnings of MRI signal changes.

Limited studies have explored the relationship between *post mortem* MRI and histology in ALS with assessment restricted to a segment of the primary motor cortex of a hemisphere in small number of cases. Reported changes include R2* hyperintensity in the middle and deep layers of the grey matter and signal alteration in the subcortical white matter of the motor cortex [9]. Histopathological evaluation of the corresponding cortical region showed microglial iron accumulation and myelin pallor in subcortical white matter [9]. Similarly, diminished contrast between grey and white matter has been observed, with altered T1 relaxation rate ratio in the motor cortex corresponding to reduced neuronal and axonal density and increased astroglial density and reactivity [10]. The study also identified comparable differences in T1 relaxation rate ratio in other cortical regions anterior and posterior to the motor cortex as a gradient suggestive of contiguous pathology spread [11–13], though with limited histological evaluation. Interestingly, no significant changes in average T2 relaxation rate ratio were detected [10]. These MRI modalities are sensitive to more than one aspect of tissue related to neuropathology. For accurate interpretation of specificity and validation of the sensitivity of such findings, multiple MRI modalities accompanied with a range of histological parameters must be considered, extending to other regions involved in disease progression.

Combining whole brain *post mortem* MRI with systematic histological evaluation representing ALS-FTD pathology propagation is a pathway to determining and validating disease specific changes in structural organisation and connectivity. Whole fixed brain MRI confer additional benefits over MRI of small segmented regions, including reduced artefacts related to MRI, preserved landmarks for topographical identification of regions of interest, and a single scan that can cover the whole brain [14]. Given the growing interest, studies have established direct correlation between *post mortem* whole brain MRI in situ with large-scale digital histology on corresponding coronal slices for evaluation of pathology and structural connectivity [15–18]. However, whole slice digital histology is costly and labour-intensive, therefore routine implementation is not feasible on a large number of cases. Interestingly, precise comparison of high-resolution whole brain *post mortem* MRI with histological analysis on strategically sampled pathologically relevant regions of interest in multiple sclerosis have also been demonstrated [14, 19].

This article outlines a universal whole brain sampling strategy suitable for systematic evaluation of differential microstructural and molecular changes associated with pathology spread in cortical, subcortical and deep white matter regions of ALS and FTD to ultimately enable accurate *post mortem* MRI-histology correlation. The proposed methodology, modified from a standard brain banking protocol, was carefully designed to selectively sample pathologically relevant regions guided by anatomical landmarks and tractography. The protocol takes into consideration various aspects of downstream co-registration requirements and quantitative histological analyses to facilitate accurate depiction of changes within the regions of interest.

## Methods

### Methodological considerations

#### Identification of neuroanatomical regions relevant to pathology spread

Identification of neuroanatomical regions that depict ALS and FTD pathology spread is crucial to establishing a universal whole brain sampling strategy by identifying clinical phenotypes and accompanying neuropathological characterisation. Clinical phenotypes of ALS are heterogeneous and the focality of symptom onset and subsequent contiguous spread of motor dysfunction are common to all diagnosed patients and indicative of a disease continuum [20]. Neuropathologically, ALS is characterised by a degree of upper and lower motor neuron loss concomitant with degeneration of their respective axonal projections, glial activation and the pathological aggregation of 43-kDa TAR DNA-binding protein (TDP-43) [21, 22]. The unique molecular feature of TDP-43 proteinopathy is reported in almost all cases of ALS and in the largest subset of FTD supporting the concept of ALS-FTD as a pathological spectrum [12, 23]. Although neuropathological evaluation is made at the “end-stage” of disease, recent studies have defined a pathological staging system based on systematic neuroanatomical distribution of TDP-43 pathology that correlated with severity of clinical phenotypes [11, 12, 24, 25].

In a system of ALS pathological ‘stages’ based on regional patterns of *post mortem* phosphorylated TDP-43 (pTDP-43) pathology, Stage 1 was defined as involving the upper motor neurons of the primary motor cortex and the lower motor neurons of the brainstem and the spinal cord [25]. From the motor cortex, Stage 2 involves pathology extended rostrally to prefrontal cortical regions of the middle frontal gyrus and caudally to the reticular formation of the brainstem, red nucleus and precerebellar nuclei. Stage 3 was defined as pTDP-43 lesions extending into the gyrus rectus and orbital gyri of the basal prefrontal cortex, post-central sensory areas of the parietal lobe, the temporal lobe and the striatum. Stage 4 involves pathological burden in the anteromedial regions of the temporal lobe and the hippocampus. Distinct pTDP-43 distribution patterns have also been proposed for behavioural variant FTD (bvFTD) [12], the most common form of FTD overlapping with ALS [26]. In bvFTD, pTDP-43 lesions first appear in the basal prefrontal cortex (orbital gyri and gyrus rectus) and amygdala in Pattern I. Involvement caudally into the prefrontal cortical regions (middle frontal gyrus, insular cortex and anterior cingulate), temporal lobe, including hippocampus, and subcortical regions striatum, thalamus, red nucleus and precerebellar nuclei of pons and medulla defines Pattern II. In Pattern III, motor and parietal cortical regions and lower motor neurons of the brainstem and spinal cord are involved. In contrast to ALS, Pattern IV in bvFTD involves pTDP-43 within the occipital pole [12].

The concept of anterograde corticofugal propagation of pTDP-43 is supported by these patterns and the presence of pTDP-43 pathology in the subcortical white matter of the affected cortical areas and the involvement of subcortical regions with substantial neocortical afferents [11, 25, 27]. Diffusion tensor imaging has been utilised to analyse white matter tracts that are likely to be involved in corresponding pTDP-43-based stages of ALS in vivo [28]. The study observed substantial differences in corticospinal tract (Stage 1), corticorubral and corticopontine tracts (Stage 2), corticostriatal pathway (Stage 3) and proximal portion of perforant pathway (Stage 4) in ALS in comparison to the control group and tract involvement corresponded with disease duration. Experimental evidence in vivo also supports involvement of axonal pathways in FTD. Diffusion tensor imaging of bvFTD showed significant degree of white matter damage to the uncinate fasciculus, inferior and superior longitudinal fasciculus, genu of corpus callosum, forceps minor and cingulum bundle [29–31]. Furthermore, the fornix was identified as a key locus of damage in bvFTD [32]. However, a more limited number of studies have validated histopathological changes in deep white matter tracts with demonstrated involvement in ALS pathology in vivo. Independent neuropathological validation demonstrated a lack of pTDP-43 pathology in corticospinal tract, corpus callosum and cingulum bundle in ALS staging [27]. Interestingly, other distinctive pathological features such as glial activation and axonal degeneration in corticospinal tract and corpus callosum have been reported previously [33–36], and callosal involvement is one of the most consistent MRI findings in ALS [37, 38]. These findings suggest that the underlying pathobiological features of neuroimaging modalities remain unclear highlighting the need for validation of MRI modalities with their respective histologic correlates at cellular and subcellular levels.

Validation of microstructural changes in regions representing pTDP-43 spreading pattern in *post mortem* brains against whole brain MRI signals requires systematic sampling of pathologically relevant regions of interest. Presented in Table 1 are the key neuroanatomical regions (cortical and subcortical grey matter; subcortical and deep white matter tracts) that (1) represent ALS and bvFTD pathology spread, and (2) are topographically recognisable with defined anatomical landmarks to facilitate systematic sampling and MRI-histology co-registration.

**Table 1:** Regions of the brain involved in pTDP-43 staging system in ALS and bvFTD

Regions of interest	ALS	bvFTD
*Grey matter*		
Precentral gyrus (motor cortex)	Stage 1	Pattern III
Brainstem		
Hypoglossal nucleus	Stage 1	Pattern III
Precerebellar nuclei/red nucleus	Stage 2	Pattern II
Middle frontal gyrus	Stage 2	Pattern II
Cingulate—anterior	Stage 2/3	Pattern II/III
Postcentral gyrus (somatosensory cortex)	Stage 3	Pattern III
Orbitofrontal cortex		
Orbital gyrus	Stage 3	Pattern I
Gyrus rectus	Stage 3	Pattern I
Striatum	Stage 3	Pattern II
Hippocampal formation	Stage 4	Pattern II
Occipital pole	Not affected	Pattern IV
*White matter tracts*		
Corticospinal tract	Stage 1*	Pattern III*
Cingulum	Not affected [27]	Affected*
Fornix	Not assessed	Affected*
Corpus callosum	Affected*	Affected*

The table highlights brain regions that have been described in the *post mortem* four stage hypothesis of pTDP-43 [12, 25], or demonstrated to be involved in the disease process in vivo

* White matter tract changes demonstrated with in vivo MR imaging, however, independent of pTDP-43 validation or in the absence of pathology [27, 39]

#### Systematic sampling and MRI-histology co-registration requirements

Standardised systematic sampling should be implemented for making valid statements about regions of interest, however, challenged by the highly folded nature of the cortex, large number of areas with variable sizes and undeniable inter-individual variability [40]. Nevertheless, maintaining reproducibility with neuroanatomical accuracy is crucial to a standardised sampling strategy in a disease specific cohort. The proposed sampling strategy considers two approaches to minimise inter-individual variability whilst maintaining neuroanatomical accuracy: (1) the use of neuroanatomical landmarks, e.g. gyri, sulci and subcortical grey matter that can be robustly identified in all brains on MRI and during cut-up of the corresponding brain, for identifying cortical and subcortical regions of interest and (2) the use of diffusion MRI tractography to guide identification and sampling of white matter tracts. These approaches enabled implementation of systematic sampling for regions of interest to provide accurate estimates of changes in them [41] and are aimed to facilitate multi-modal alignment and co-registration of 2-dimensional (2-D) histological images on 3-dimensional (3-D) MR volumes [42, 43].

**Fig. 1:**
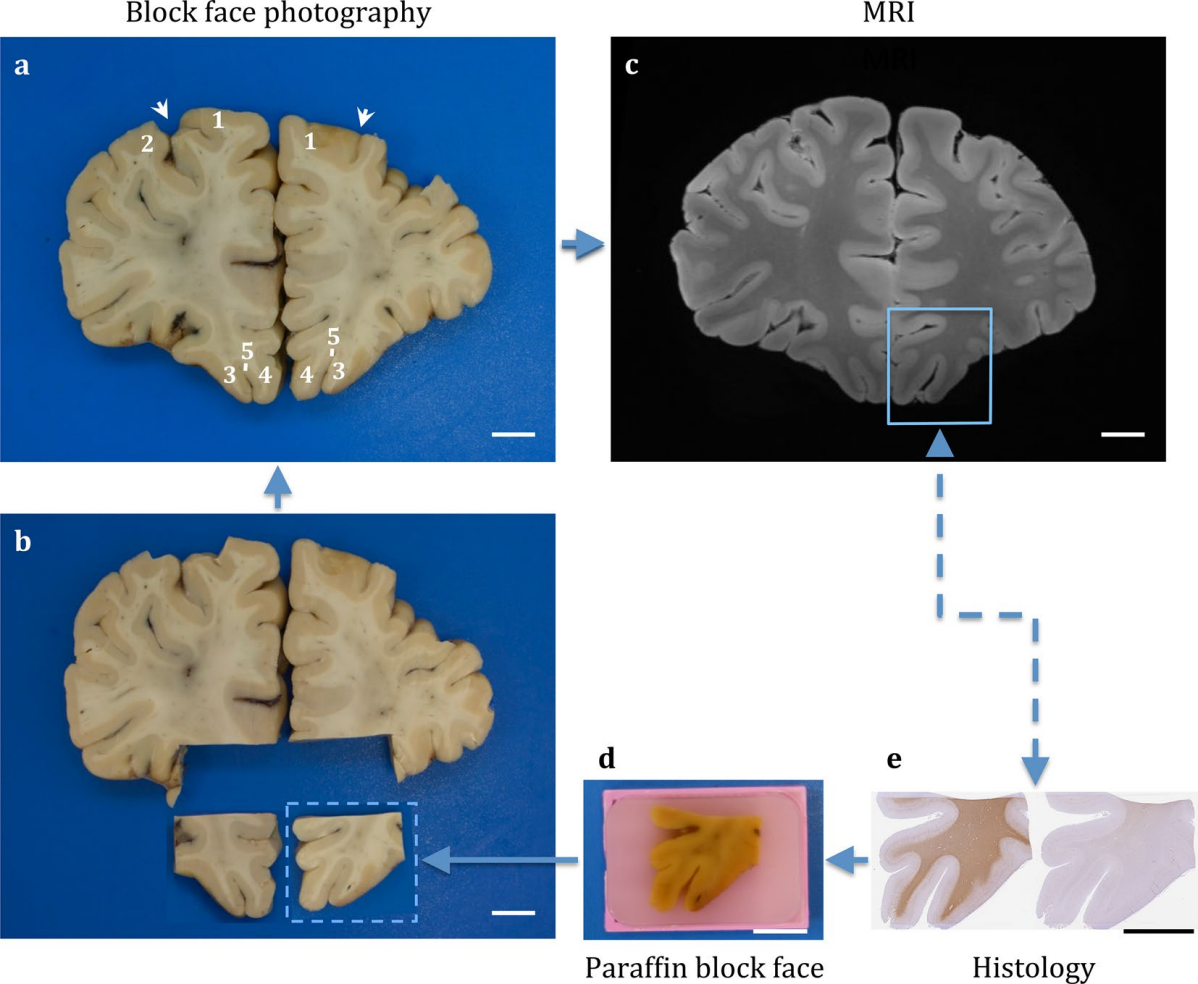
Block face photography and digital histology pipeline for MRI-histology co-registration. Brain digitally photographed at various stages of block face sampling including intact brain, coronal slices (**a**) and extracted regions of interest (**b**). These images are coupled with images of trimmed surface paraffin embedded blocks (**d**) and serial digital histology images (**e**) for mapping (dotted arrow) on MRI modalities (**c**). Solid arrows represent registrations. Annotations—1: superior frontal gyrus, 2: middle frontal gyrus, 3: medial orbital gyrus, 4: gyrus rectus, 5: olfactory sulcus, white arrow: superior frontal sulcus. Scale bar = 1 cm

Direct MRI-histology correlation, in vivo and ex vivo, is generally made difficult by the heterogeneity in characteristics on images from the two modalities in addition to the deformation and damage caused by histological processing [42]. Although substantial distortions are likely to occur at the time of fresh brain extraction and during fixation, geometric deformations also occur during MRI scanning due to the brain being stabilised in a close-fitting container resulting in twisting or compression. Subsequent serial sampling of the fixed brain following scanning, histological sectioning of the brain regions into 2-D slices and staining would often introduce tears, rips, folds, missing pieces of tissue, artefacts, debris, uneven staining gradient and displacement of anatomical landmarks such as gyri and sulci from the original 3-D geometry [40, 43, 44]. In addition, tissue shrinkage as a result of chemical processing for paraffin embedding needs to be taken into account. Several studies have explored registration of histology to MR with digital photographs of block faces acquired during sampling, in addition to stained sections, to serve as an intermediate modality for alignment and reconstruction on a 3-D volume [40, 43–47]. To enable accurate co-registration whilst taking into consideration structural deformations and damage, the proposed sampling strategy is coupled with digital photography pipeline (Fig. 1) at various stages of block face sampling and histological processing: (1) coronal slices (2) sampled regions of interest and their remnants, and (3) trimmed surfaces of paraffin blocks with (4) stained serial histological sections.

### Tissue preparation and MR imaging

*Post mortem* tissues were obtained from the Oxford Brain Bank. Fixed whole brains were drained of formalin and immersed in 3 M™ Fluorinert™ (FC-3283) for susceptibility matching (Fluorinert has a similar susceptibility to tissue, however, no signal) in a closed fitting plastic mould and imaged with a Siemens 7 T scanner using 32-channel receive, single-channel transmit RF coil for 48 h. Magnetic resonance imaging acquisition consisted of a set of protocols that provides a variety of contrasts (Table 2), including structural scans, relaxography (T1, T2), susceptibility-weighted contrast and diffusion weighted steady-state free precession (DW-SSFP) for more specific measures of white matter [48–50]. Following MRI, brain drained of Fluorinert™ and returned to formalin in preparation for sampling.

**Table 2:** Whole brain MRI protocol parameters

*a. DW*-*SSFP*	
Lines per TR	3
q value (cm^−1^)	300
b_eff_ (s/mm^2^)	5150
TE/TR (ms)	21/28
Resolution (mm)	0.85 × 0.85 × 0.85
Flip angle (°)	24 and 94
Number of directions	120 per flip angle
Bandwidth (Hz/pixel)	393
Duration 1 volume (min:s)	5:50
Total duration (h:min:s)	24:30:00
*b. Structural 3*-*D*—*Trufi*	
TE/TR (ms)	4.09/8.16
Resolution (mm)	0.25 × 0.25 × 0.27
Flip angle (°)	30
Phase cycling (°)	0, 90,180,270
Averages (per PC)	2
Bandwidth (Hz/pixel)	393
*c. T1*-*map turbo spin*-*echo*	
TE/TR (ms)	14.0/1000
Resolution (mm)	1.0 × 1.0 × 1.0
Flip angle (°)	90 and 180
Averages	1
Bandwidth (Hz/pixel)	130
Inversion times (ms)	60, 120, 240, 480, 935
*d. T2*-*map turbo spin*-*echo*	
TEs (ms)	25, 38, 50, 63, 76
TR (ms)	1000
Resolution (mm)	1.0 × 1.0 × 1.0
Flip angle (°)	90 and 180
Averages	1
Bandwidth (Hz/pixel)	130
*e. Susceptibility weighted imaging multi*-*gradient echo*	
TEs (ms)	2, 8.6, 15.2, 21.8, 28.4, 35
TR (ms)	38
Resolution (mm)	0.5 × 0.5 × 0.6
Flip angle (°)	15
Number of repetitions	4
Bandwidth (Hz/pixel)	650

### MR image processing pipelines

Structural datasets obtained with the 3-D – TRUFI protocol were averaged by calculating the root-mean-square of the individual phase-cycled scans. The T2-mapping protocol utilised a voxel-wise, signal-weighted pseudoinverse of the linearised temporal signal evolution from the T2 turbo spin-echo protocol. An identical process was adopted to generate T2*-maps, fitting to the magnitude component of signal evolution from the susceptibility weighted imaging (SWI) protocol. The T1-mapping protocol utilised a non-linear least-squares fit to the signal evolution from the T1 turbo spin-echo protocol. All fitting was performed using the NumPy and SciPy toolboxes in Python [51, 52].

Processing of the diffusion data obtained via the DW-SSFP imaging protocol utilised modified forms of DTIFIT and BEDPOSTX from the FSL toolbox [53–55], to account for the DW-SSFP [48, 56], dual flip-angle datasets. All co-registration between and within imaging modalities was performed using FSL-FLIRT [57, 58].

Phase datasets from the SWI protocol were processed to generate quantitative susceptibility maps in MATLAB (The MathWorks, Inc.), original phase images were first unwrapped using a Laplacian-based method [59], the unwrapped phase images were subsequently filtered using V-SHARP algorithm [60] to remove the background field, quantitative susceptibility maps were finally generated using STAR-QSM algorithm from the STI Suite toolbox [61].

### Sampling strategy for regions of interest

Brains were prepared separately for sampling initially by carefully removing the meninges, photographed at several planes and dimensions were measured prior to dissection. Brain stem and cerebellum were removed from the cerebrum by knife section across the cerebral peduncle at the level of the 3rd cranial nerve (oculomotor nerve) in a plane perpendicular to the brainstem and aqueduct. Specific regions of interest were then sampled in both hemispheres as described in the following sections.

#### Motor and somatosensory cortex

**Fig. 2:**
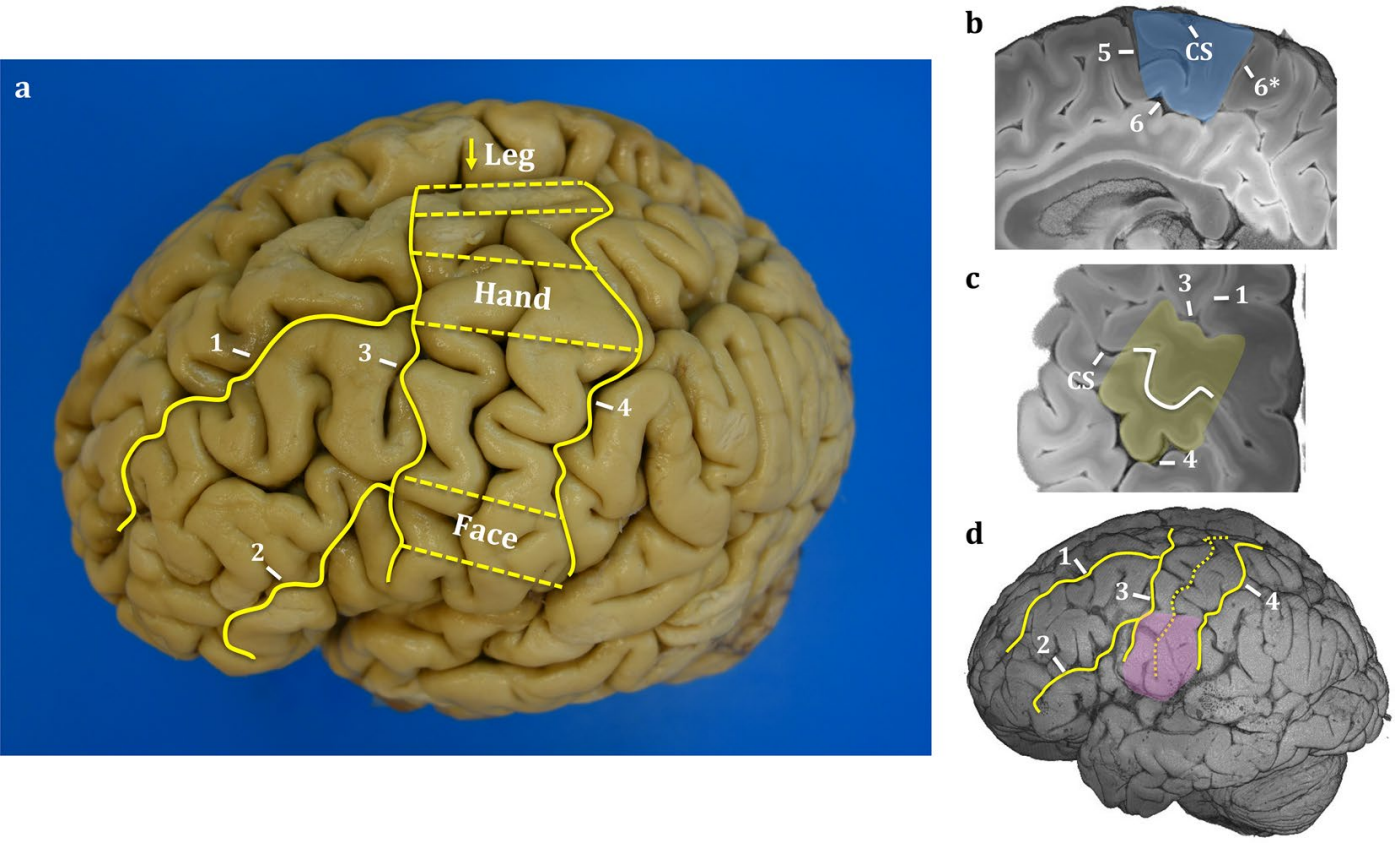
Motor and somatosensory cortex leg, hand and face region sampling. Identification of motor and somatosensory cortical regions of interest by the sulci (solid yellow lines) on the dorsal and lateral surfaces (**a**). Dashed yellow lines (**a**) represents the leg, hand and face regions excised for analysis. Corresponding MR structural images of paracentral lobule (**b**), hand knob (**c**) and lateral face (**d**) areas are highlighted in blue, yellow and pink, respectively. The hand knob recognised by the inverted omega on the central sulcus of the MR structural image (**c**). Major sulci were used as anatomical landmarks for guided sampling. Annotations—1: superior frontal sulcus, 2: inferior frontal sulcus, 3: pre-central sulcus, 4: post-central sulcus, 5: paracentral sulcus, 6: cingulate sulcus, 6*: marginal segment of the cingulate sulcus, *CS* central sulcus, arrow: interhemispheric fissure where leg region sampled on the paracentral lobule

The motor cortex (ALS Stage 1) on the precentral gyrus is demarcated by the anatomical borders the precentral sulcus (anteriorly) and the central sulcus (posteriorly) on the dorsolateral surface. Somatosensory cortex was identified in the post-central gyrus posterior to the central sulcus. Sampling of the motor and somatosensory cortex was restricted to clinicopathologically relevant regions defined by the anatomical landmarks (Fig. 2):

1. Leg area was represented on the medial surface of the paracentral lobule at the banks of the interhemispheric fissure.
2. Motor hand area was distinguished by the hand knob (shaped as an inverted omega).
3. Face area was identified on the precentral gyrus lateral to the intersection of inferior frontal sulcus and precentral sulcus.

Approximately 1 cm blocks of tissue perpendicular to the central sulcus were dissected out at the leg area, middle hand knob region and face area to include pre-central gyrus, corresponding post-central gyrus, central sulcus and subcortical white matter (Fig. 2). Care was taken to ensure that blocks were sampled at a reasonable depth to maintain integrity of pre- and post-central gyri together with the central sulcus on the same block (to facilitate the identification of the M1/S1 border, which is in the depth of the sulcus).

#### Coronal slices

**Fig. 3:**
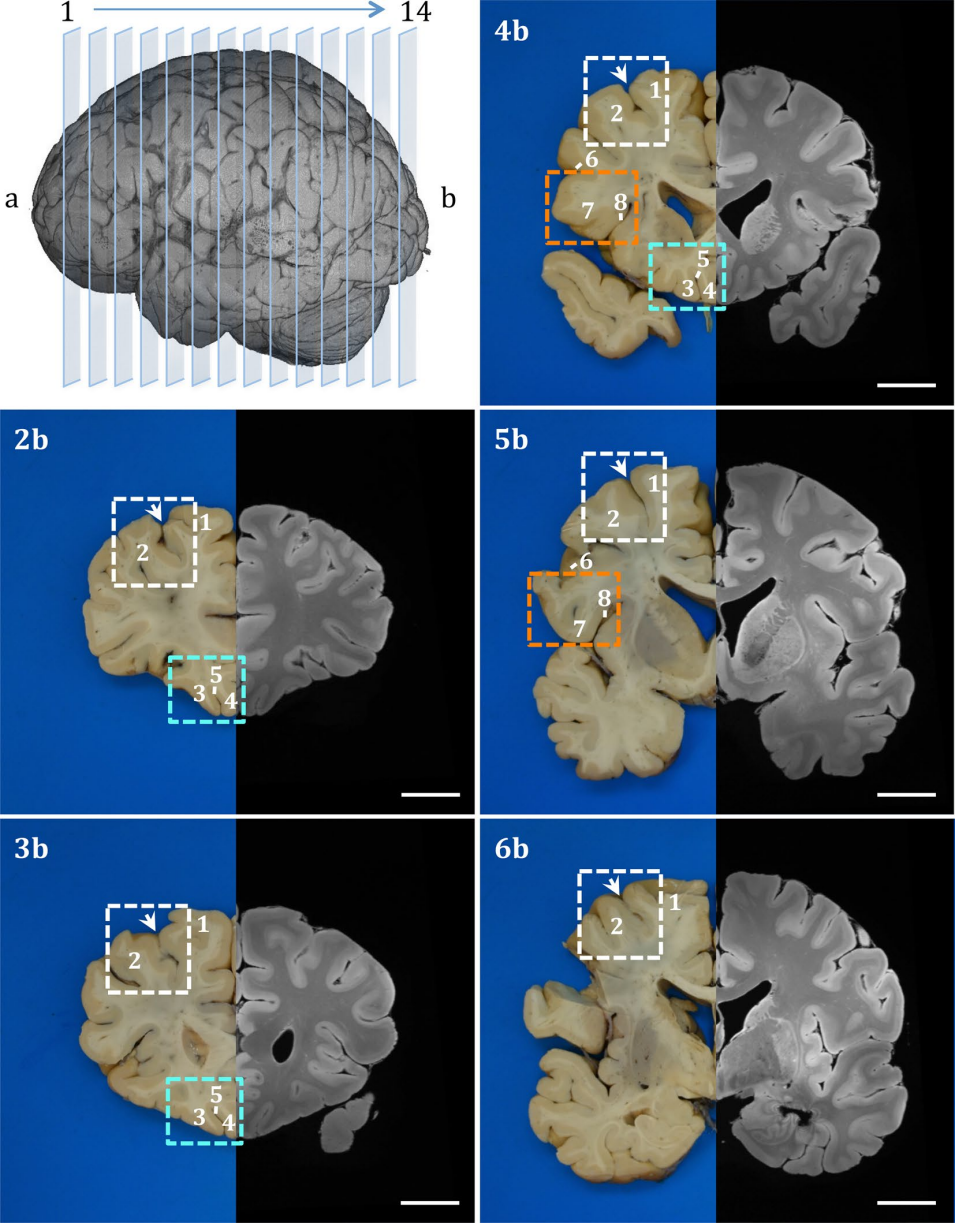
Systematic sampling strategy for the cortical regions of the frontal lobe. The 3-D MRI image on top left-hand corner represents slicing of the brain on coronal plane. Each slice numbered consecutively in rostral-caudal axis and labelled as anterior (**a**) or posterior (**b**) when digitally photographed. Images **2b**–**6b** show gross brain coronal slices (posterior plane) with mirrored structural MR image from the left hemisphere. Key neuroanatomical regions middle frontal gyrus (white rectangle), orbitofrontal cortex (orbital gyri and gyrus rectus: turquoise rectangle) and inferior frontal gyrus (orange rectangle) were sampled in serial coronal slices systematically with their accompanying subcortical white matter. Major sulci were used as landmarks for region and anatomical boundary identification. Annotations—1: superior frontal gyrus, 2: middle frontal gyrus, 3: medial orbital gyrus, 4: gyrus rectus, 5: olfactory sulcus, 6: inferior frontal sulcus, 7: inferior frontal gyrus, 8: circular insular sulcus, arrow: superior frontal sulcus. Scale bar = 2 cm

Following extraction of the primary motor and somatosensory cortical regions, brains were sliced in a coronal plane through the mammillary bodies perpendicular to the longitudinal axis of the forebrain. Subsequent coronal slices, at 1 cm intervals, were made anterior and posterior to the initial cut (Fig. 3). Anterior and posterior surfaces of each slice were photographed prior to sampling of the following regions of interest.

#### Middle frontal gyrus

The middle frontal gyrus is involved in Stage 2 and Pattern II of ALS and bvFTD, respectively. It forms part of the dorsolateral prefrontal cortex involved in regulation of attention, executive function and working memory that have been reported to be deficient in ALS patients without dementia [62–64]. The middle frontal gyrus is one of the largest regions in the prefrontal cortex located between the superior frontal sulcus, inferior frontal sulcus and pre-central sulcus [65]. It cytoarchitectonically constitutes Brodmann areas (BA) 6, 8, 9, 46 and 10 along its caudal-rostral axis [65, 66].

The middle frontal gyrus was identified in coronal slices lateral to the anatomical landmark superior frontal sulcus and sampled at a reasonable depth (~ 2 cm) to include subcortical white matter, and the adjacent superior frontal gyrus and sulcus (Fig. 3). Sampling was carried out in serial coronal slices rostral to the precentral gyrus extending to the anterior most slice that clearly defines the superior frontal sulcus.

#### Orbital gyri and gyrus rectus

The orbital gyri and gyrus rectus, involved in the later stages of ALS (Stage 3) and in the earliest stage of bvFTD (Pattern I), lie in the basal surface of the frontal lobe. The gyrus rectus extends longitudinally in the medial border of the basal surface. The medial orbital gyrus is situated immediately lateral to the gyrus rectus and demarcated by the olfactory sulcus. The gyrus rectus and the medial orbital gyrus were sampled in rostral-caudal axis in serial coronal slices (Fig. 3).

#### Inferior frontal gyrus (Broca’s area)

Impairment in language and syntactic processing has been implicated in ALS patients [67–70]. Pathological changes in Broca’s area, associated with speech production and a wide range of language and communication related functions [71–73], have been reported in ALS with and without other FTD features [67]. Broca’s motor speech area and its homologue in the right hemisphere occupy the caudal part of the inferior frontal gyrus demarcated by the precentral sulcus posteriorly, inferior frontal sulcus superiorly and the lateral fissure inferiorly [65]. The area is further delineated macroscopically by the opercular and triangular gyri corresponding to BA 44 and 45 [71, 74]. Broca’s area was sampled from the inferior frontal gyrus, including the depth of the circular insular sulcus in two coronal slices (~ 2 cm) rostral to the precentral sulcus (Fig. 3).

#### Corticospinal tract, thalamus and striatum

**Fig. 4:**
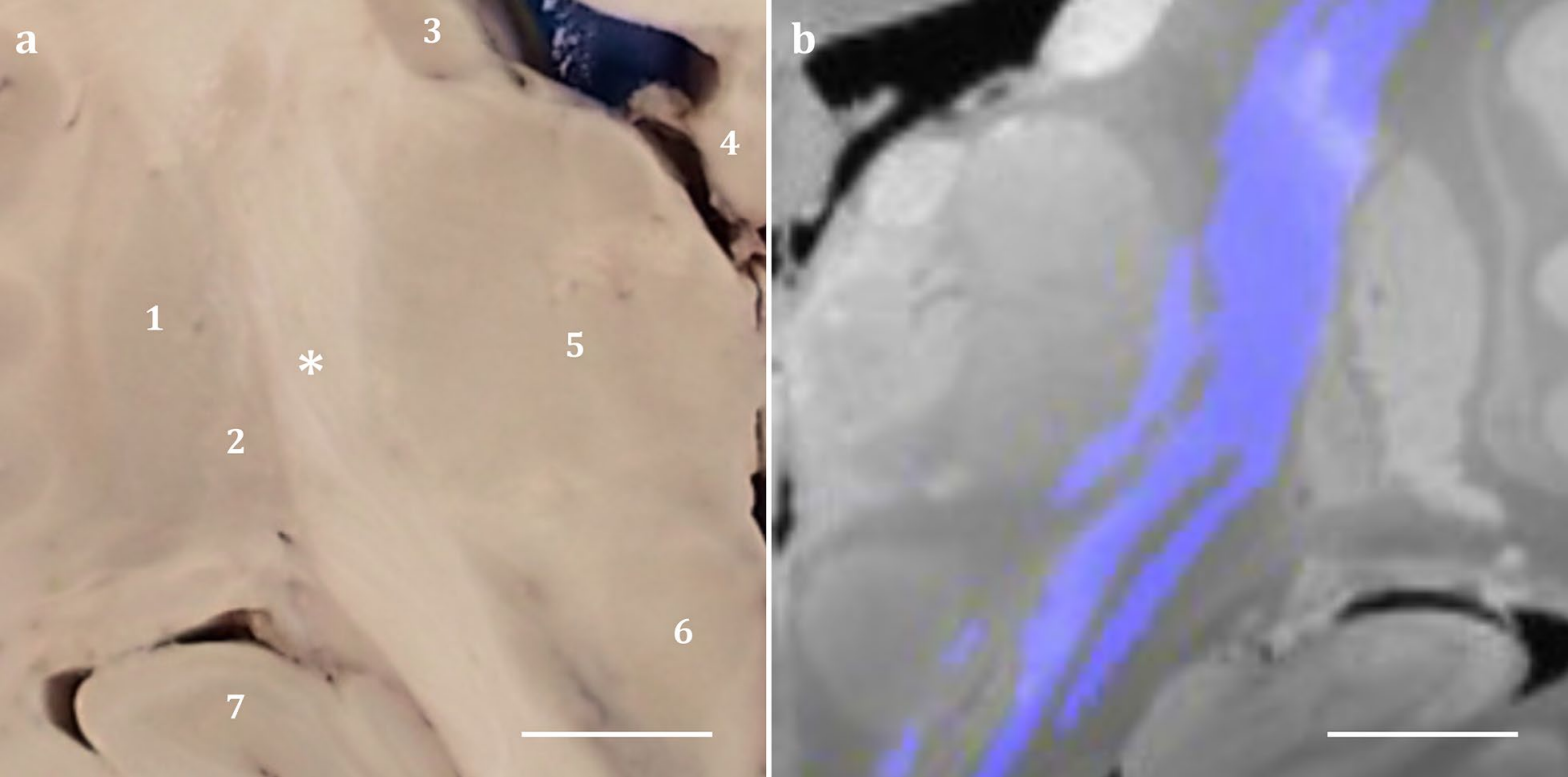
Tractography guided identification of the corticospinal tract. The slice (**a**) that best represents the corticospinal tract (purple) was identified with tractography (**b** mirrored MR image). Annotations—1: putamen, 2: external globus pallidus, 3: caudate, 4: body of fornix, 5: thalamus, 6: red nucleus, 7: hippocampus, *posterior limb of internal capsule. Scale bar = 1 cm

Identification of the corticospinal tract was guided by diffusion tractography of fibres originating from the leg area of paracentral lobule, and hand and face regions of the precentral gyrus (Fig. 4). The corticospinal projections that descend through the posterior limb of the internal capsule were sampled with the surrounding thalamus and the lentiform nucleus. Following sampling of the corticospinal tract, the remaining basal ganglia and internal capsule were sampled in anterior coronal slices.

#### Hippocampal formation

The hippocampal formation of the dorsomedial temporal lobe, involved in the final stage of ALS and representing Pattern II of bvFTD, constitutes the hippocampus with dentate and adjacent cortical regions extending to the parahippocampal gyrus. The parahippocampal gyrus is located inferior to the hippocampus between the hippocampal fissure and collateral sulcus. The hippocampal formation, identified by its unique topographical organisation, was sampled in consecutive coronal slices superior to the collateral sulcus.

#### Corpus callosum, cingulate gyrus and paracallosal cingulum

The cingulate gyrus is situated on the medial surface of the brain extending from the rostral genu area and dorsally to the body of the corpus callosum and terminating ventral to the splenium [75]. The white matter underlying the cingulate cortex presents the paracallosal cingulum bundle. The cingulate gyrus, inferior to the cingulate sulcus and subparietal sulcus, was sampled serially in the coronal plane together with the corpus callosum.

#### Fornix

The fornix, which lies immediately inferior to the corpus callosum, is an arch shaped white matter tract carrying efferent fibres from the hippocampus primarily into the mammillary bodies. The fornix is divided into three regions named crus, body and column along the caudal-rostral axis [76]. The crus of the fornix, an extension of the fimbria of the hippocampus, from both hemispheres arches below the splenium of the corpus callosum forming the body of the fornix. The fibre bundle then descends at the rostral end, at the level of the anterior commissure, dividing into the columns of the fornix. Given the shape and compact nature of the fornix, sampling is restricted to macroscopically distinguishable regions such as the body of the fornix (Fig. 4), which can be sampled together with the thalamus or the corpus callosum (refer to previous sections). Presentation of the crus and column of the fornix is dependent on the plane and the level of coronal cut and therefore sampled only when the regions can be macroscopically recognised on the coronal plane.

#### Brainstem and cerebellum

The brainstem was sampled perpendicular to its longitudinal axis to include multiple levels of the midbrain, pons and medulla at 5 mm intervals.

**Fig. 5:**
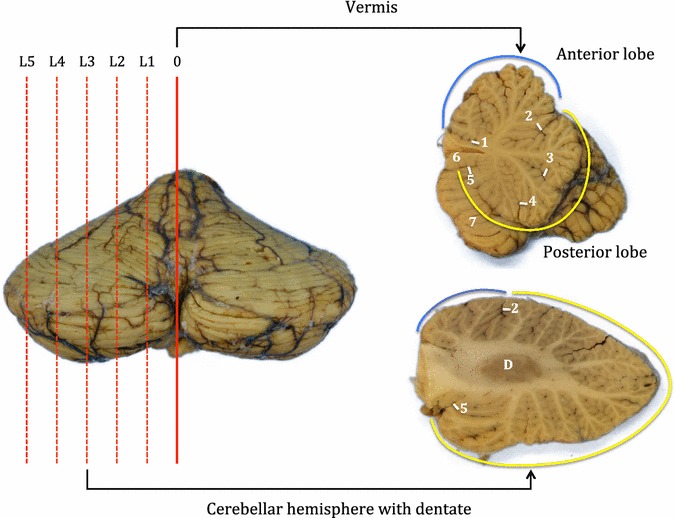
Sampling of the cerebellum. Cerebellum sampled on sagittal plane by bisecting through midline of the vermis (level 0) and at subsequent multiple levels (L1–L5) into the cerebellar hemisphere at a set thickness. Annotations—1: precentral fissure, 2: primary fissure, 3: prepyramidal fissure, 4: secondary fissure, 5: posteriolateral fissure, 6: nodulus, 7: tonsil, *D* dentate, blue line: anterior lobe, yellow line: posterior lobe

Cerebellar folial pattern and the anatomy of the lobules are best studied in serial parasagittal sections [77, 78]. The cerebellum was bisected sagittally in the midline through the vermis and subsequent slices were cut through the cerebellar hemispheres at 5–7 mm thickness (Fig. 5). Whole sagittal slices were sampled from vermis to cerebellar hemisphere, including the dentate nucleus.

#### Internal controls

In addition to regions that are involved in ALS and associated FTD, regions predicted to have little or no involvement in the disease process were sampled to serve as internal controls. This is a crucial part of the protocol because in our experience any prospective *post mortem* ultra-high-field MRI study of the human brain is affected by a number of interindividual variables that are difficult to control. However, the strength of a whole brain MRI approach with extensive histological sampling means that an intraindividual calibration of both MRI and histology data is possible via the inclusion of areas that remain unaffected by disease.

The primary visual cortex (BA 17), situated in the medial surface of the occipital lobe, which can be clearly identified by the stria Gennarii at the banks of the calcarine fissure, was sampled with the adjacent secondary visual area (BA 18) and subcortical white matter. The forceps major are radiations of commissural fibres arising from the splenium of the corpus callosum that connects the occipital lobes. Guided by tractography, the forceps major was sampled along the medial wall of the posterior horn of the lateral ventricle and superior to calcar avis on a coronal slice posterior to splenium.

#### Other regions

Representative blocks of regions that are not described here were sampled as per standard brain banking protocol for diagnostic purposes.

### Histological processing, immunohistochemistry and quantitative digital image analysis

All sampled tissues were processed for paraffin embedding. Briefly, formalin fixed samples were dehydrated in a series of graded ethanol solutions (70–100%), cleared in xylene and embedded in paraffin wax. Subsequently, paraffin block surfaces were trimmed and photographed prior to acquiring serial sections at 6–10 μm thickness for histology (10 μm thickness for pTDP-43 and 6 μm for other stains). Immunohistochemistry was performed with a range of primary antibodies to detect myelin, inflammation (microglia and astroglia activation), iron, neurofilaments and pTDP-43 (Table 3), and visualised using DAKO EnVision Systems and counterstained with haematoxylin. Whole slides were digitised at high resolution (×40 objective for pTDP-43 and ×20 objective for all other stains) with Aperio ScanScope®AT Turbo (Leica Biosystems) high-throughput slide scanner.

**Table 3:** Primary antibodies

Antibody	Host	Staining dilution	Supplier	Cat. no.	Remarks	References
CD68 (PGM-1)	Mouse	1:50	DAKO	M0876	Marker for activated microglia and macrophages	[79, 81]
Ferritin	Rabbit	1:3000	Sigma	F5012	Iron storage protein	[79, 83, 84]
GFAP	Rabbit	1:2000	DAKO	Z0334	Astroglia marker	[79, 80]
Iba1	Rabbit	1:1000	WAKO	019-19741	Microglia marker	[81]
PLP	Mouse	1:1000	Bio-Rad	MCA839G	Major myelin protein	[82]
pTDP-43 (S409/410 -clone 9-11)	Mouse	1:40 000	Cosmo Bio	TIP-PTD-M01	Phosphorylated TDP-43	[27, 85, 86]
SMI-311	Mouse	1:1000	Biolegend	837801	Pan neuronal marker that recognise non-phosphorylated epitopes of neurofilament heavy and medium chains. Stains pyramidal neurons	[87–89]
SMI-312	Mouse	1:2000	Biolegend	837901	Pan axonal neurofilament marker that recognise phosphorylated epitopes of neurofilament heavy and medium chains	[87, 90]

**Fig. 6:**
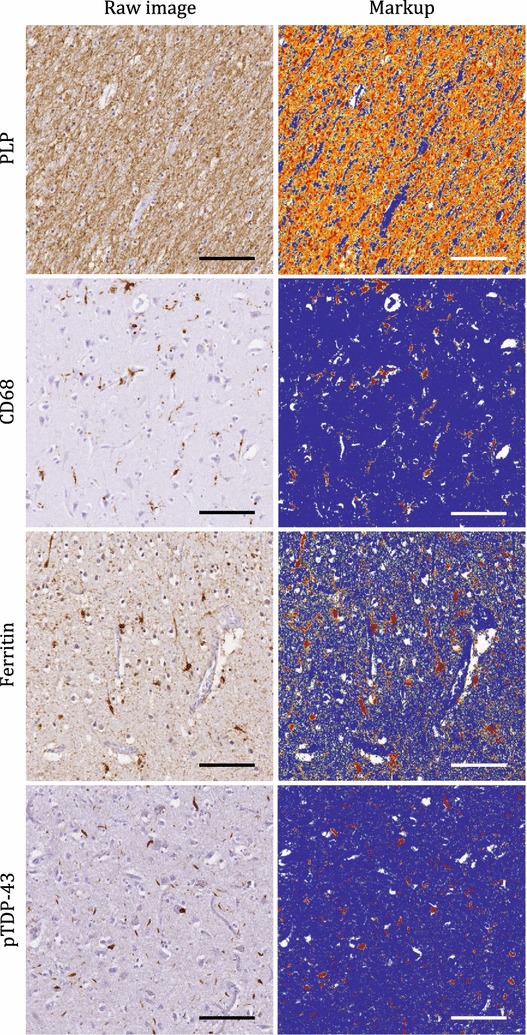
Quantitative digital histology image analysis. The high-resolution digital histology images represent the motor cortex subcortical white matter stained for PLP and grey matter stained for CD68, ferritin and pTDP-43 in ALS. Colour deconvolution for positive staining (brown) generates markup images demonstrating unstained pixels = white, negative stained pixels = blue, weak positive pixels = yellow, medium positive pixels = orange and strong positive pixels = red. Scale bar = 100 μm

The relative burden of pathology for immunohistochemical staining in regions of interest was analysed in digital images using Aperio Colour Deconvolution algorithm (version 9.1, Leica Biosystems). Colour deconvolution facilitates stain separation by calibration of colour vectors for each stain (brown immunostain and blue haematoxylin) to generate measurements of selected positive colour channel and intensity thresholding to exclude non-specific background staining. Algorithm input parameters were calibrated and separate threshold for each stain was established by finding a threshold that yielded robust results in at least 10 randomly selected structurally distinct regions [91]. Regions of interests were outlined manually on each image in Aperio ImageScope based on general cytoarchitectural morphology and density differences (Fig. 6), while histological artefacts such as staining artefacts, debris, folds, tears and air bubbles in regions of interest were outlined to exclude from analysis. The colour deconvolution algorithm was applied to quantify the stained area fraction (area of positive staining over total analysis area, Fig. 6) for myelin proteolipid protein (PLP), glial activation (ionised calcium binding adaptor molecule 1: Iba1, cluster of differentiation 68: CD68, glial fibrillary acidic protein: GFAP), ferritin and neurofilaments (SMI-311 and SMI-312), and validated by a neuropathologist. Aperio image analysis algorithms have been used in previous studies to quantify the amount of immunostaining underlying neuropathological changes [92–98] and the fraction of positively stained pixels in an area were reported with demonstrated sensitivity to packing density, size and number of cells and their processes [92, 93, 99].

The colour deconvolution algorithm, as described above, was utilised to quantify the burden of pTDP-43 in manually outlined regions and reported as stained area fraction. Similar algorithms have been used previously for quantification of pathological TDP-43 load [100, 101]. Classification of pTDP-43 phenotype was based on cellular localisation and cortical distribution and categorised into neuronal cytoplasmic inclusions, dystrophic neurites, neuronal intranuclear inclusions, and/or glial inclusions by a neuropathologist according to the accepted criteria [102–104].

### Registration

**Fig. 7:**
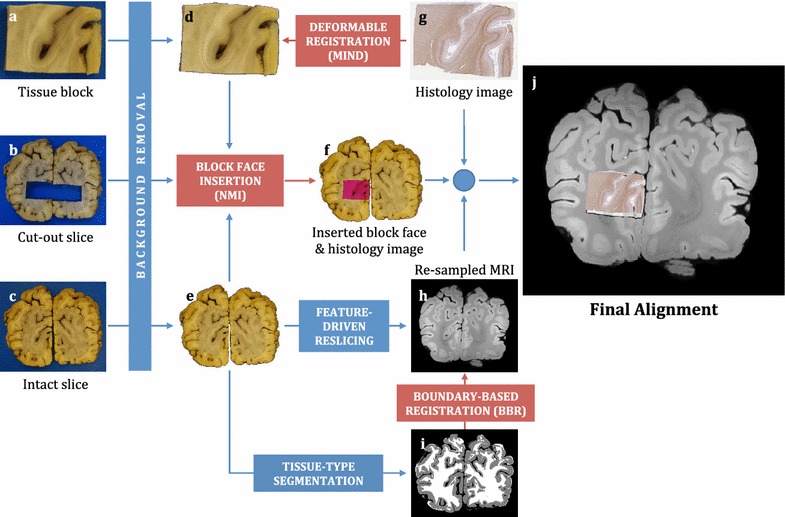
Overview of MRI-histology co-registration approach. **a** Photograph of tissue block (block face). **b** Photograph of coronal brain slice after the excision of tissue blocks. **c** Photograph of intact coronal brain slice. **d** Same as **a** after removing the blue background. **e** Same as **c** after removing the blue background. **f** Photograph of intact brain slice with inserted block face. Block insertion is a rigid-body registration that uses normalised mutual information (NMI [58]) as a cost function. The histology image will, later on, be transformed to fit the region of interest defined by this block. **g** Histology image that is registered to the block face using non-linear, deformable registration (cost function: MIND [106]). **h** Slice of the MR volume that was re-sampled along a curvilinear surface to represent the anatomical features of **e**. **i** Tissue-type segmentation of **e**. The boundary between white matter and grey matter is registered to the same boundary in **h** using boundary-based registration (cost function: BBR [108]). **j** Final alignment of the histology to the resampled MR volume (only one slice is shown)

As illustrated in Fig. 7, the strategy for registration involves several separate stages. All of these are encapsulated in our actively developed software tool (EMMA for Efficient Microscopy–MRI Alignment) [105] that we ultimately intend to release publicly. The first stage in the registration process involves separating the foreground and background for the photographs (Fig. 7a–c). In the second stage the photographed tissue block (or block face) is located within the photograph of the cut-out slice, which we call block face insertion (Fig. 7f). The insertion step itself is a rigid-body registration between the block face (Fig. 7d) and the photograph of the intact brain slice (Fig. 7e). It is informed by the photograph of the cut-out slice (Fig. 7b, after background removal) to minimise the chance of an erroneous insertion. The third stage involves registering the histology images (Fig. 7g) with the block face photograph (Fig. 7d), which are likely to involve distortions and tears associated with slicing sections from the block, but have the same edges. To overcome the resolution gap (0.5 μm/pixel vs. 50 μm/pixel), histological images are first sub-sampled to the resolution of the photographs. Tissue shrinkage and large-scale distortions are addressed by an initial affine transformation of the histological images. Finally, small-scale distortions are gradually compensated by smoothly deforming the histological images (Fig. 7g) to follow the edges of the block face (Fig. 7d). To make the registration work across modalities, both the histological images (Fig. 7g) and the block face (Fig. 7d) are represented using the Modality Independent Neighbourhood Descriptor (MIND) [106]. MIND was developed for the purpose of cross-modality image registration, and its robustness has already been demonstrated in multiple applications [106], including the registration of brain MR images to histology [107]. The fourth stage involves registering the digital photograph of the intact coronal section (Fig. 7e) to the 3-D MR image of the hemisphere prior to slicing. This registration needs to deal with the relative deformations involved with the brain being placed into the scanner and then cut into slices, meaning that the cut face need not be a simple plane in the 3-D image but would in general be a slightly curved surface. Therefore the MR volume is first re-sampled (Fig. 7h) parallel to a (curvilinear) surface that best represents the anatomical features in the photograph, then grey-white matter boundary information is obtained from a tissue-type segmentation (Fig. 7i to drive the boundary-based registration (BBR) process [108]. The final stage then combines these various stages together, to obtain a non-linear registration of the histology image to the appropriate portion of the 3-D MR image (Fig. 7j). Each stage involves specific challenges but is a lot simpler than a direct registration of the histology image to the 3-D MR image, as a direct registration has far fewer anatomical features to work with as well as very large changes in the type of intensity contrast.

## Results

**Fig. 8:**
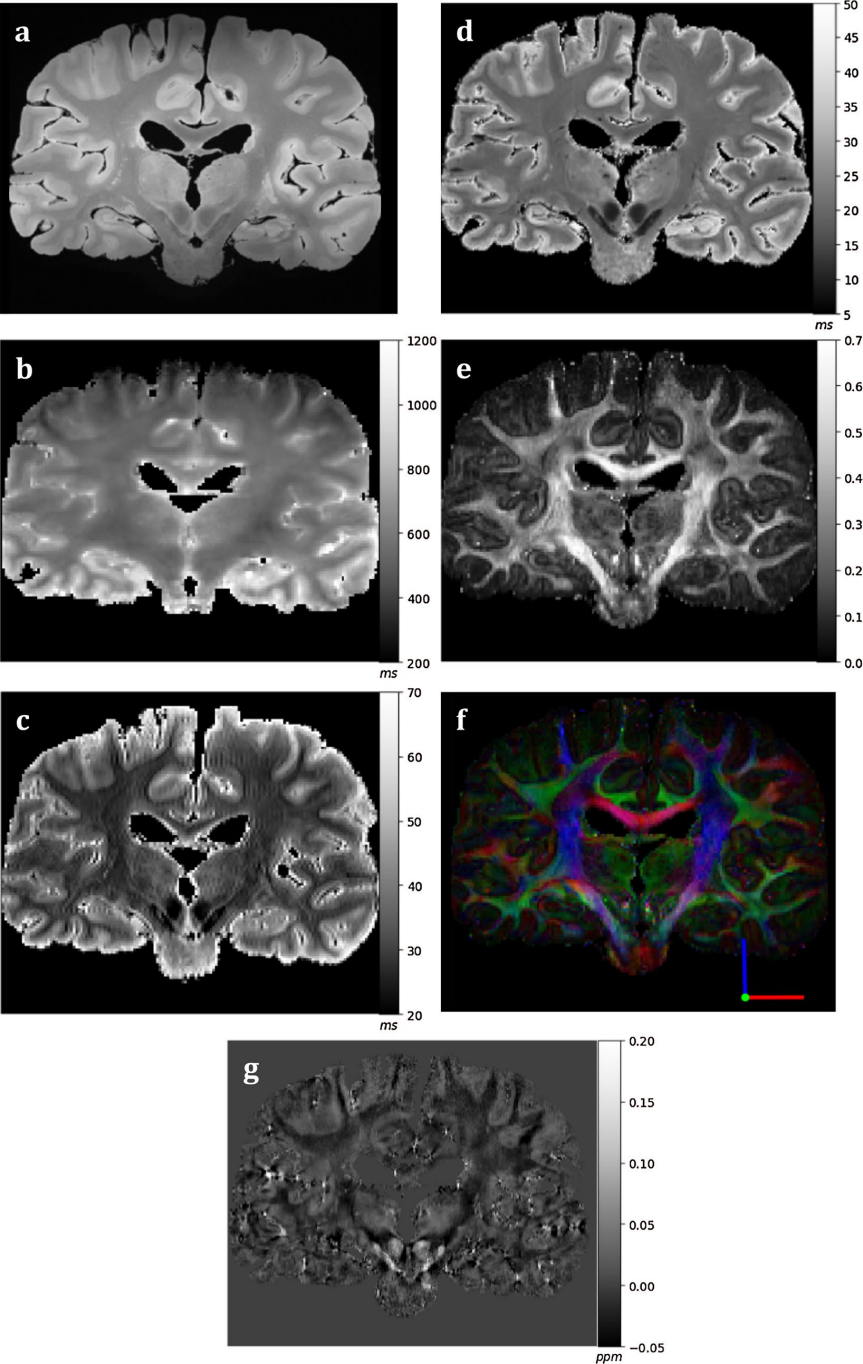
Processed MRI datasets from an individual brain. The coronal MR images display a structural map (**a**), T1-map (**b**), T2-map (**c**), T2*-map (**d**), FA map (**e**), principal diffusion direction weighted by the FA (**f**) and susceptibility map (**g**). The calibration bars for the T1-map (**b**), T2-map (**c**) and T2*-map (**d**) indicate the T1, T2 and T2* values (in ms), respectively. The calibration bar in the FA map (**e**) indicates the fractional anisotropy. The key at bottom right of **f** indicates colour orientation of the principal diffusion direction. The calibration bar in the susceptibility map (**g)** indicates the susceptibility in parts-per-million (ppm)

**Fig. 9:**
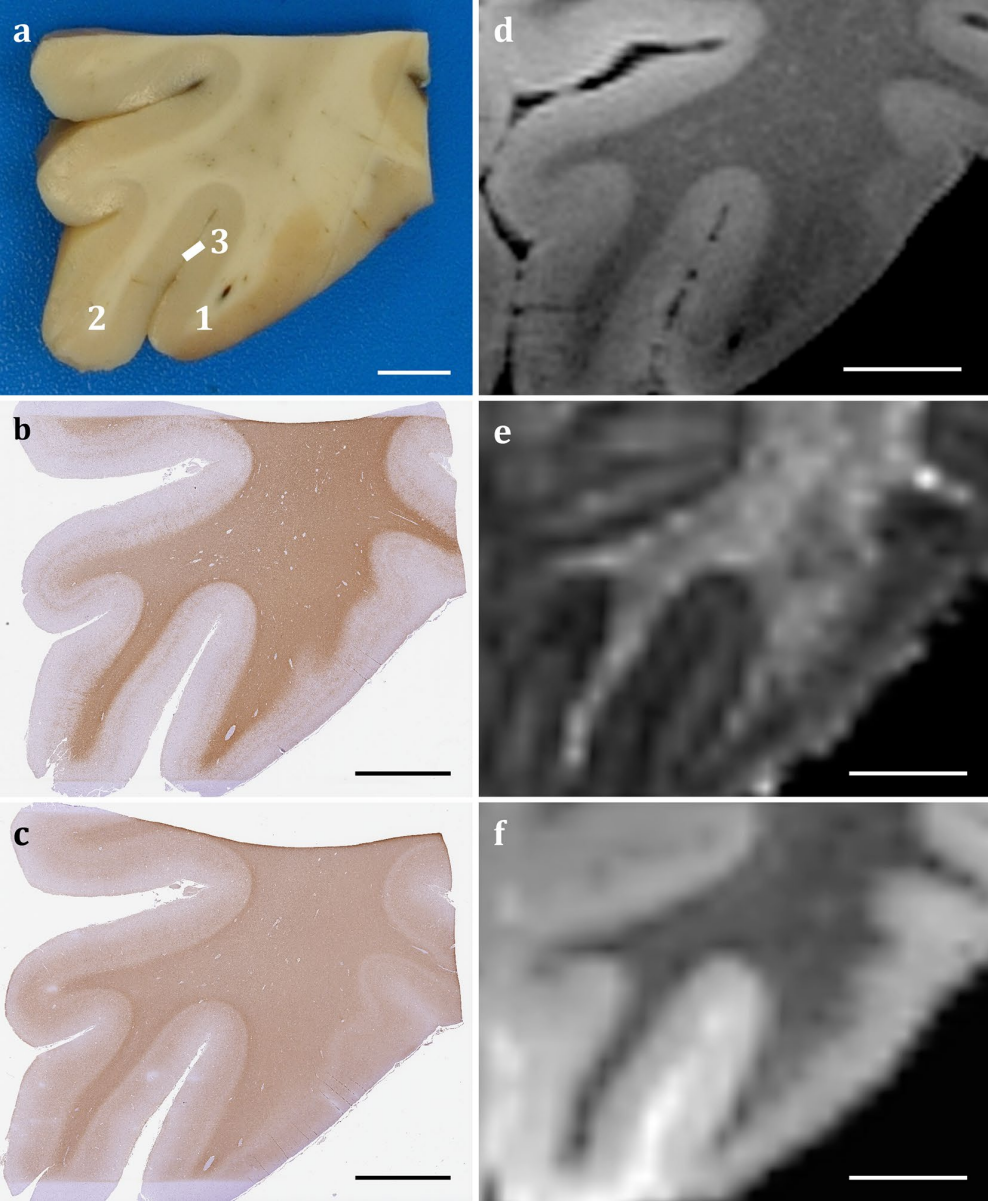
Example of histology-MRI comparison of the orbitofrontal cortex. Histology sections stained for PLP (**b**) and SMI-312 (**c**) were obtained from the block face (**a**) sampled from the orbitofrontal cortex. High staining intensity of myelin PLP and SMI-312 neurofilament was evident in the subcortical white matter with clear contrast between the cortical grey and white matter boundary. Corresponding plane from structural (**d**), FA (**e**) and MD (**f**) MR images showed changes in signal intensity that were comparable to histology. Annotations—1: medial orbital gyrus, 2: gyrus rectus, 3: olfactory sulcus. Scale bar = 5 mm

**Fig. 10:**
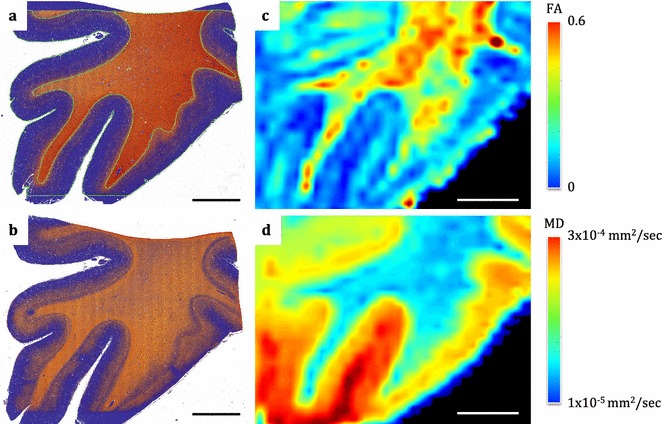
Histology and MRI quantitative maps. Colour deconvolution markup histology images stained for PLP (**a**) and SMI-312 (**b**) demonstrates negative stained pixels = blue, weak positive pixels = yellow, medium positive pixels = orange and strong positive pixels = red. The PLP markup image shows manual outline of the grey matter and the subcortical white matter (green line). Images on the right column demonstrate quantitative FA (**c**) and MD (**d**) maps from the corresponding MRI plane and their calibration bars with quantitative values. Scale bar = 5 mm

Preliminary results from each processed MRI modality for a brain are displayed in Fig. 8. The individual modalities display distinctive anatomical contrast and unique information about the underlying tissue composition and microstructure. Furthermore, an example of comparison between multiple histological stains and MRI modalities on a mapped plane from the normal appearing orbitofrontal cortex and its subcortical white matter is demonstrated in Fig. 9. Serial histology sections stained for axonal myelin (PLP) and neurofilament content (SMI-312) were mapped to the corresponding MRI plane (structural and diffusion weighted) that represents the region of interest, spared from pathology, from an ALS patient (ALS 1) (Fig. 9). Qualitative evaluation of the stains demonstrates high myelin and neurofilament stain density in the white matter compared to the grey matter and distinct grey/white matter boundary (Fig. 9). The grey scale structural, fractional anisotropy (FA) and mean diffusivity (MD) images showed comparable contrast in signal intensity that corresponded with histology. Similar observations were made in quantitative FA and MD maps (Fig. 10) indicating sensitivity of these MRI modalities to multiple components of axonal microstructure. Further validation is required with additional stains that represent other microstructural features and should be compared with other MRI modalities for accurate interpretation of these findings.

**Fig. 11:**
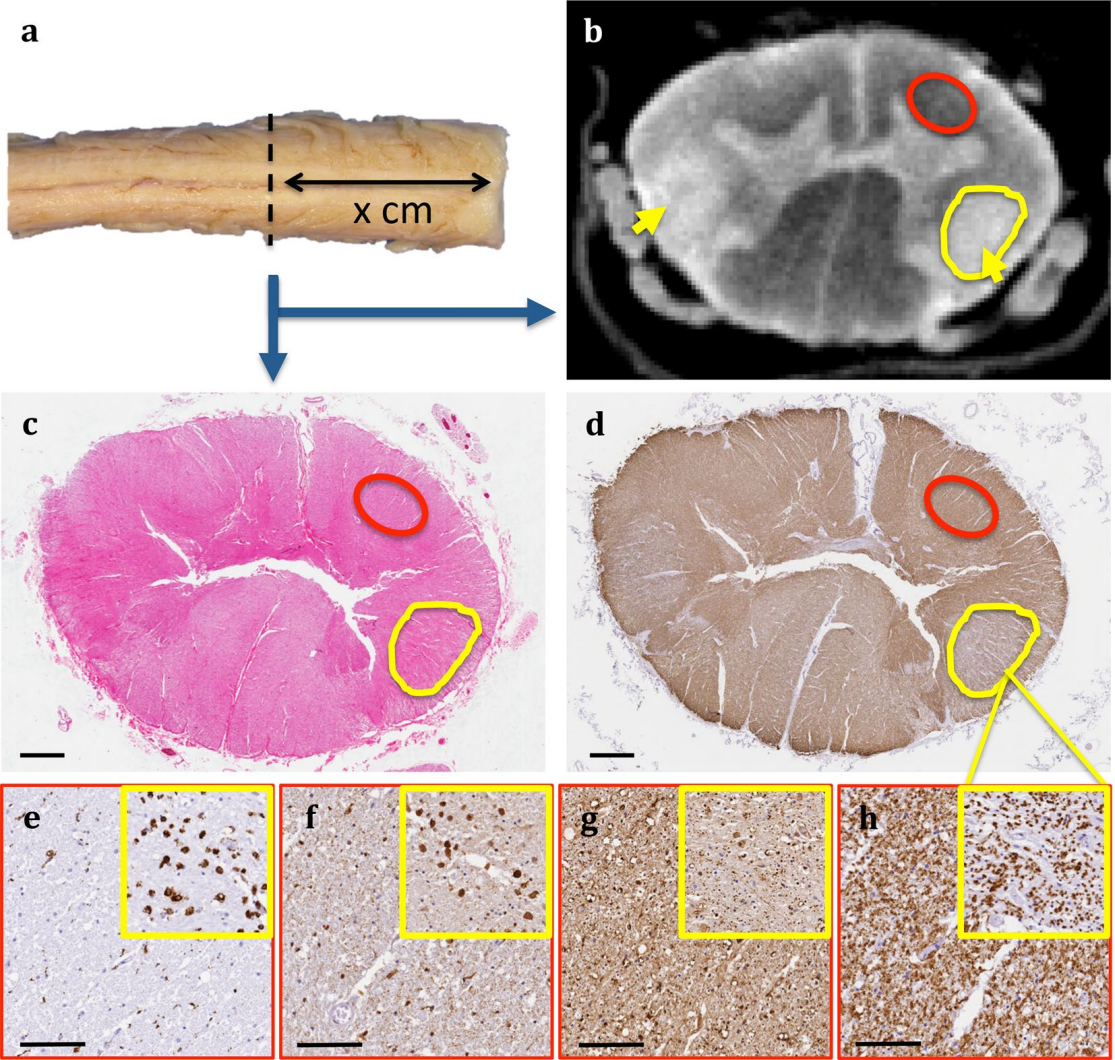
Qualitative MRI and histological evaluation of the lateral corticospinal tract degeneration in the spinal cord from an ALS patient. MRI and histology was assessed at the same plane (**a**). The structural MRI (**b**) shows lateral corticospinal tract hyperintensity (yellow arrow and circle) compared to normal appearing white matter region (red circle). Images **c**, **d** represent low magnification snapshots of the spinal cord stained with haematoxylin and eosin and PLP, respectively. Images in the bottom row demonstrate qualitative comparison between normal appearing white matter in red boxes and lateral corticospinal tract in yellow box inserts at ×20 objective magnification for CD68 (**e**), ferritin (**f**), SMI-312 (**g**) and PLP (**h**) stains. Quantitative MRI and histology analysis outputs for these regions are presented in Table 4. Scale bar for **c**, **d** = 1 mm. Scale bar for figures **e**–**h** and corresponding inserts = 100 µm

Proof of concept examining the influence of disease specific microstructural pathological changes on multiple MRI modalities was demonstrated in the *post mortem* spinal cord from an ALS patient (ALS 2). The MRI acquisition of the spinal cord consisted of a set of protocols (Additional file 1) for structural scan, T1- and T2-mapping, and DW-SSFP. The structural MRI showed lateral corticospinal tract hyperintensity along the scanned segment of the spinal cord (Additional file 2). The latter MRI signal change was concomitant with the degeneration of the lateral corticospinal tract histologically demonstrated with marked axonal myelin and neurofilament loss together with heightened inflammatory response (CD68) on a segment of the cervical cord (Fig. 11 and Table 4). Quantitative evaluation of the same region on the corresponding MRI plane demonstrated significant decrease in average FA value (*P* < 0.001) and significant increase in average MD, T1 and T2 values (*P* < 0.001) in comparison to the normal appearing white matter region (Table 4).

**Table 4:** Quantitative MRI and histology evaluation of the lateral corticospinal tract of the spinal cord in ALS

Regions	MRI				Histology			
	FA	MD (×10^−5^ mm^2^/s)	T1 (ms)	T2 (ms)	PLP	SMI-312	CD68	Ferritin
Lateral Corticospinal Tract	0.17 ± 0.03	11.23 ± 1.19	714.8 ± 31.4	34.2 ± 2.11	0.379	0.356	0.044	0.061
Normal appearing white matter	0.19 ± 0.07	7.95 ± 2.52	672.1 ± 23.6	30.9 ± 1.31	0.696	0.606	0.016	0.036
*P* value	< 0.001	< 0.001	< 0.001	< 0.001	NA	NA	NA	NA

Quantitative MRI and histological data from the lateral corticospinal tract of the spinal cord was compared to the normal appearing white matter region as outlined in Fig. 11. Magnetic resonance imaging data were quantified per voxel in the regions of interest with two-tailed *t* test and reported as mean ± standard deviation. The regions of interest within the lateral corticospinal tract and normal appearing white matter were generated from structural MR image and applied to each modality that were analysed. The MR data were calculated in 276 voxels with 100 mm^2^ cross sectional area in the lateral corticospinal tract and in 269 voxels with 97.6 mm^2^ cross sectional area in the normal appearing white matter. On the corresponding histology plane, a single section was stained for each representative microstructural marker and the stained area fraction (area of positive staining over total analysis area) was calculated

**Fig. 12:**
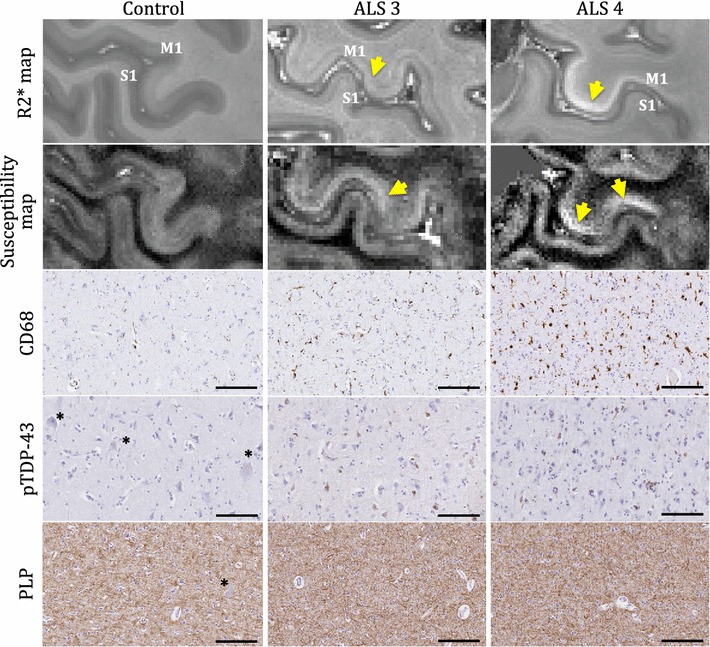
Qualitative comparison of signal changes in R2* and susceptibility maps and histological evaluation in the primary motor cortex of an age matched control and 2 ALS patients. MRI and histology was assessed in the hand knob region of the primary motor cortex. Evaluations were made specifically in the grey matter at the posterior bank of the precentral gyrus. The first and second row show R2* (inverse of T2*) and susceptibility map in axial plane for a control and 2 ALS patients. Increase in cortical hyperintensity (yellow arrow) is evident in ALS 3 and ALS 4 in comparison to the control. Images in the subsequent rows demonstrate the relative burden of CD68, pTDP-43 and PLP. Quantitative histology analysis outputs are provided in Additional file 3. Annotations—M1: motor cortex, S1: sensory cortex, *Betz cells. Scale bar for CD68 and PLP images = 200 µm. Scale bar for pTDP-43 images = 100 µm

The burden of pathology in the hand knob area of the primary motor cortex grey matter was compared between 2 ALS patients, with varying degree of pathology, and a control (Fig. 12). Qualitative comparisons were made in MRI signal changes in R2* and QSM maps that have been previously demonstrate to be sensitive microglial iron accumulation [7, 9]. Compared to the control, ALS 3 shows moderate diffused hyperintense signal changes in both R2* and susceptibility maps. ALS 4 shows the strongest hyperintense signal change in R2* and susceptibility maps. Upon qualitative and quantitative histological evaluation of the same region, the intensity in signal changes corresponds to the relative burden of CD68 pathology in all cases, with lowest in the control and the highest in ALS 4 (Fig. 12 and Additional file 3). Quantitative pTDP-43 burden appears to be slightly higher in ALS 4 (Additional file 3), however its relationship to CD68 pathology is unclear and further evaluations are warranted. Interestingly, cortical myelin content in the ALS cases was similar to that of the control (Fig. 12 and Additional file 3). Patient data for ALS and control cases reported are presented in Table 5. The pTDP-43 staging for each ALS case was classified according to the accepted criteria [104].

**Table 5:** Patient data and neuropathological diagnosis

Tissue ID	Age (years)	Sex	Neuropathological evaluation	TDP-43 stage
Control	61	M	Control	–
ALS 1	68	M	ALS–TDP type	Stage 4
ALS 2	76	M	ALS with FTD–TDP type	Stage 4 and Pattern III
ALS 3	71	M	ALS–TDP type	Stage 4
ALS 4	61	M	ALS–TDP type	Stage 2

**Fig. 13:**
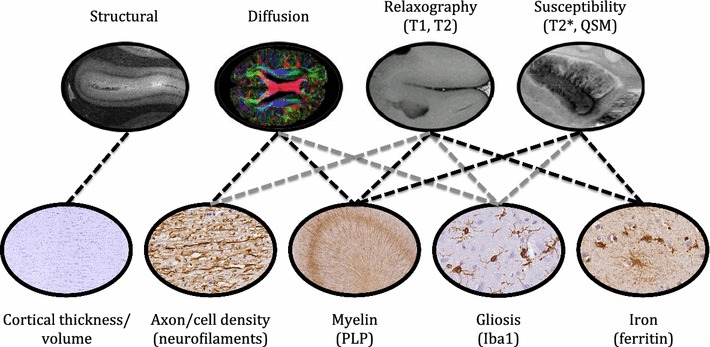
Predicted sensitivity of MRI modalities to microstructural properties of the neural tissue. Structural, diffusion, relaxography and susceptibility MRI (top row) modalities can be influenced by several aspects of tissue microstructure. The bottom row represents microstructural features and an example of their corresponding histological stain. Predicted relationships between MRI and histology are indicated by dashed lines. The black dashed lines indicate microstructural features that can strongly influence MR signal [7, 107, 119–124]. The grey dashed lines represent other microstructural features that can potentially influence the MR signal (directly or indirectly) and/or to a lesser degree [98, 121]. It should also be noted that any MR measure might show correlation with other microstructural characteristics that are not highlighted in the image

These findings from the lateral corticospinal tract degeneration of the spinal cord demonstrate multiple MRI modalities that are influenced by several candidate features of microstructural pathological changes. The findings from the motor cortex indicate that this protocol is suitable for the investigation of subtle microstructural changes. However, more robust validation of specificity and sensitivity of these MRI modalities should be extended to a larger cohort and are ideally demonstrated in numerous brain regions with heterogeneous degree of pathological changes. Therefore, the proposed methodology for systematic histological evaluation of pathology propagation in *post mortem* whole brain in ALS and validating the pathophysiological correlates with MRI signal changes would be ideal to uncover the specificity and sensitivity of MRI measures (Fig. 13).

## Discussion

The extensive landmark based systematic whole brain sampling strategy is aimed for the accurate study of pathological propagation in ALS through identification of regions that represent the proposed four stage pTDP-43 spreading pattern with neuroanatomical accuracy. In addition to the sampling of cortical and subcortical grey matter, tractography can be used to guide accurate sampling and histological analysis of white matter tracts of interest, particularly where these tracts are not demarcated by an anatomical landmark. Systematic sampling of regions of interest was combined with a digital photography pipeline of sampling and histological processing to facilitate alignment and registration required for validation of sensitivity and specificity of MRI modalities to microstructural changes.

One of the attributes of the proposed protocol is sequential sampling of whole regions of interest with distinct topographical features and neuroanatomical landmarks. However, partial sampling was considered for several regions due to size limitations, lack of defined landmarks and adequate representation of pathology spread. Specific examples of the latter include the large middle frontal gyrus, without defined anterior boundary, where sampling was restricted to its dorsal portion immediately lateral to the superior frontal sulcus. In addition, only the main clinicopathologically relevant segments of the primary motor and somatosensory cortex (pre-central and post-central gyri) were sampled. Sampling of the fornix was limited to the body and fimbria, whereas crus and column region extraction on the coronal plane was restricted by slice thickness and variation in cutting angle of coronal slices for macroscopic identification. In such events, accurate MRI-histology correlation in regions of interest may be challenged by the limited availability of histology. Therefore, findings should be interpreted with careful consideration and reported with neuroanatomical accuracy.

Defining white matter tracts on histological images are generally guided by anatomical boundaries derived from location-based histological atlases. Unlike DTI tractography, which facilitates 3-D delineation and visualisation of multiple white matter tracts simultaneously [109], histological delineation of multiple white matter tracts of interest with a common anatomical trajectory on 2-D plane is difficult. For example, the posterior limb of the internal capsule located between the pallidum and thalamus contain corticospinal, corticobulbar and corticopontine projections and thalamic radiations along the superior-inferior axis [110, 111]. Tractography can be utilised for identification of the histological slice that best depict the tract of interest whilst taking into consideration the other adjacent tracts that may or may not be involved in the disease process. If more than one tract of interest is identified on a single stained histology section, they should be clearly demarcated prior to histological analysis using tractography-derived data. Furthermore, interpretation and reporting of findings should consider the possibility of multiple tracts being represented on analytical planes.

The visual cortex and forceps major are regions where ALS associated degenerative changes are not anticipated; they have been used previously as internal controls or reference regions [10, 28, 112]. Conversely, some in vivo imaging studies have reported changes in functional connectivity [113, 114], grey matter atrophy, cortical thinning and altered glucose metabolism in occipital cortex of individuals with ALS independent of visual impairment [115–117]. These findings have not been validated ex vivo, although it has been reported that pTDP-43 pathology may propagate into occipital lobe in the final stages of bvFTD [12]. Growing evidence recognise ALS as a multisystem disorder and given the complex connectivity within the brain, identification of regions that are entirely spared of pathological involvement is challenging. Therefore, identification of internal control regions should be based on the likelihood of no or minimal involvement in the disease process, however their validity should be assessed individually for each case with histological analysis. It is further essential that molecular and morphological features of the internal control regions are comparable with aged-matched controls with no known neurological disease.

Quantitative characterisation of digital histology images has been recognised as important for advances in high-throughput pathological diagnosis and research, and minimises subjective biases generally expected from traditional qualitative or semi-quantitative (arbitrary rating-scale-based) assessments. Such analytical outputs from histology images provide a comparable platform to correlate against quantitative MR analyses, with the potential to provide an interpretation of MRI-based measures with improved biological specificity. However, the proposed quantitative digital image analysis tool is limited by its inability to discern false positive staining intensity gradient, due methodological challenges, from true positive staining. It is therefore critical to ensure that staining quality is consistently maintained (via batch processing) and all images should be assessed manually to recognise any false positive staining and overall quality prior to analysis.

Accurate alignment of a histology region of interest on the MRI plane is necessary for making direct correlations between the modalities. However, variation in slice plane orientations and spatial resolution in histology and MRI images can have impact on alignment. A typical histology section of 6 µm thickness imaged at ×20 objective magnification has an in-plane resolution of 0.502 µm/pixel. By comparison, our MR images are of lower resolution with voxel size ranging from 0.25 mm × 0.25 mm × 0.27 mm for the structural scans to 1.00 mm × 1.00 mm × 1.00 mm for the T1-and T2-maps. Alignment of smaller regions of interest that are clearly identifiable within a histology image may not be feasible on an MRI plane. Conversely, it is also important to recognise that MRI signal from larger 3-D volume may not always be reflected on a single 2-D histology section [118].

The feasibility of the proposed methodology to better interpret MRI measurements with biological specificity is supported by our preliminary findings in a small number of ALS cases. The lateral corticospinal tract degeneration in the spinal cord of an ALS patient accompanied statistically significant changes in multiple MRI modalities that corresponded with clear axonal degeneration, myelin loss, microglial activation and iron accumulation. Furthermore, the intensity of signal change in R2* and susceptibility maps of the grey matter of the primary motor cortex corresponded primarily with the degree of microglial activation in two ALS cases and a control with comparative changes to pTDP-43 burden. However, the relationship between the CD68 and pTDP-43 expression and their influence (combined and individual) on MR signal is unclear. No difference was observed in myelin content. Previous studies have reported individual microstructural features that can strongly influence a specific MR signal [7, 107, 119–124], however validation of these modalities against multiple molecular and structural features were generally not considered. Recent evidence support the concept that changes to neural microstructure are almost always never isolated to one structural feature and are generally accompanied with several molecular and microstructural changes that can influence the MR signal to a lesser degree [98, 121]. Therefore, it is the ultimate aim of our project to identify which MRI modality best maps onto which specific neuropathological feature (see Fig. 13). We aim to achieve this through extensive evaluation of multiple regions involved in ALS and FTD pathology spread coupled with histological analysis of multiple microstructural changes that can influence the MR signal in a large cohort.

## Conclusion

The proposed landmark based systematic whole brain sampling strategy of pathologically relevant regions of interest is feasible for routine implementation in a high-throughput manner for the study of disease propagation and direct MRI-histology correlation in ALS. Together with quantitative image analysis and robust registration, this sampling approach facilitates acquisition of large-scale histology datasets for accurate comparisons with quantitative MRI data. This protocol aims to elucidate the relationship of MRI signal changes with underlying pathophysiology. Furthermore, the general principles of this protocol such as identification of regions of interest, systematic sampling, digital photography pipeline for registration and histology image analysis, can be extended to other ex vivo neuroimaging studies with histology correlation.

## Additional files

**Additional file 1.** Spinal cord MRI protocol parameters.

**Additional file 2.** Structural MRI from thoracic to cervical region of the spinal cord from an ALS patient.

**Additional file 3.** Quantitative histological data for the primary motor cortex grey matter at the hand knob.

## Abbreviations

2-D: 2-dimensional
3-D: 3-dimensional
ALS: amyotrophic lateral sclerosis
BA: Brodmann area
BBR: boundary-based registration
bvFTD: behavioural variant frontotemporal dementia
CD68: cluster of differentiation 68
DTI: diffusion tensor imaging
DW-SSFP: diffusion weighted steady-state free precession
FA: fractional anisotropy
FTD: frontotemporal dementia
GFAP: glial fibrillary acidic protein
Iba1: ionised calcium binding adaptor molecule 1
MD: mean diffusivity
MIND: modality-independent neighbourhood descriptor
MR: magnetic resonance
MRI: magnetic resonance imaging
NMI: normalised mutual information
PLP: proteolipid protein
pTDP-43: phosphorylated 43-kDa TAR DNA-binding protein
SWI: susceptibility weighted imaging
TDP-43: 43-kDa TAR DNA-binding protein
