# Supplementary material for: Dissecting the pathobiology of altered MRI signal in amyotrophic lateral sclerosis: A post mortem whole brain sampling strategy for the integration of ultra-high-field MRI and quantitative neuropathology
Source: BMC Neurosci. 2018 Mar 13;19:11. doi: 10.1186/s12868-018-0416-1 (PMC5848544; doi:10.1186/s12868-018-0416-1)
Supplement: Supplementary file 1 — Additional file 1. Spinal cord MRI protocol parameters. [file 12868_2018_416_MOESM1_ESM.docx]

***Additional file 1:***

File format: .docx

Title of data: Spinal cord MRI protocol parameters

| a. DW-SSFP |  |
| --- | --- |
| Lines per TR | 1 |
| q value (cm^-1^) | 300 |
| b_eff_ (s/mm^2^) | 6000 |
| TE / TR (ms) | 21/28 |
| Resolution (mm) | 0.6 x 0.6 x 0.6 |
| Flip angle (˚) | 26 |
| Number of directions | 120 |
| Bandwidth (Hz/pixel) | 141 |
| Duration 1 volume (min:sec) | 9:49 |
| Total duration (hr:min:sec) | 20:39:00 |
| b. STRUCTURAL  Proton-density weighted spin-echo |  |
| TE / TR (ms) | 13/3500 |
| Resolution (mm) | 0.1 x 0.1 x 1.5 |
| Averages (per PC) | 2 |
| Bandwidth (Hz/ pixel) | 230 |
| c. T1-map  Turbo spin-echo |  |
| TE / TR (ms) | 11.0 / 1000 |
| Resolution (mm) | 0.5 x 0.5 x 0.5 |
| Flip angle (˚) | 90 and 180 |
| Averages | 1 |
| Bandwidth (Hz/ pixel) | 183 |
| Inversion times (ms) | 25, 50, 100, 200, 400, 800 |
| d. T2-map  Turbo spin-echo |  |
| TEs (ms) | 11, 22, 33, 44, 55, 66 |
| TR (ms) | 1000 |
| Resolution (mm) | 0.5 x 0.5 x 0.5 |
| Flip angle (˚) | 90 and 180 |
| Averages | 1 |
| Bandwidth (Hz/ pixel) | 183 |
