# Supplementary material for: Dissecting the pathobiology of altered MRI signal in amyotrophic lateral sclerosis: A post mortem whole brain sampling strategy for the integration of ultra-high-field MRI and quantitative neuropathology
Source: BMC Neurosci. 2018 Mar 13;19:11. doi: 10.1186/s12868-018-0416-1 (PMC5848544; doi:10.1186/s12868-018-0416-1)
Supplement: Supplementary file 3 — Additional file 3. Quantitative histological data for the primary motor cortex grey matter at the hand knob. [file 12868_2018_416_MOESM3_ESM.docx]

***Additional file 3:***

File format: .docx

Title of data: Quantitative histological evaluation in the hand knob of the primary motor cortex.

|  | **CD68** | **pTDP-43** | **PLP** |
| --- | --- | --- | --- |
| **Control** | 0.008 | 0.000 | 0.476 |
| **ALS 3** | 0.018 | 0.003 | 0.539 |
| **ALS 4** | 0.047 | 0.004 | 0.433 |

Quantitative values are reported as stained area fraction.
